# Combined quantification of procalcitonin and HLA-DR improves sepsis detection in surgical patients

**DOI:** 10.1038/s41598-018-30505-7

**Published:** 2018-08-10

**Authors:** Raquel Almansa, Silvia Martín, Marta Martin-Fernandez, María Heredia-Rodríguez, Esther Gómez-Sánchez, Marta Aragón, Cristina Andrés, Dolores Calvo, Jesus Rico-Feijoo, Maria Carmen Esteban-Velasco, Luis Mario Vaquero-Roncero, Alicia Ortega, Estefania Gómez-Pesquera, Mario Lorenzo-López, Iñigo López de Cenarruzabeitia, Diana Benavides, Jaime López-Sanchez, Cristina Doncel, Carmen González-Sanchez, Esther Zarca, Alberto Ríos-Llorente, Agustín Diaz, Elisa Sanchez-Barrado, Juan Beltran de Heredia, Jose Maria Calvo-Vecino, Luis Muñoz-Bellvís, Jose Ignacio Gomez-Herreras, César Aldecoa, Eduardo Tamayo, Jesus F. Bermejo-Martin

**Affiliations:** 1 Group for Biomedical Research in Sepsis (Bio∙Sepsis), Hospital Clínico Universitario de Valladolid/IECSCYL, Avda Ramón y Cajal 3, 47005 Valladolid, Spain; 2 Anesthesiology and Reanimation Service, Hospital Universitario Río Hortega, Calle Dulzaina, 2, 47012 Valladolid, Spain; 3 Anesthesiology and Reanimation Service, Hospital Clínico Universitario de Valladolid, Avda Ramón y Cajal 3, 47005 Valladolid, Spain; 4 Clinical Analysis Service, Hospital Clínico Universitario de Valladolid, Avda Ramón y Cajal 3, 47005 Valladolid, Spain; 5 Department of General and Gastrointestinal Surgery, Hospital Universitario de Salamanca, Salamanca, España; Instituto de Investigación Biomédica de Salamanca (IBSAL), Universidad de Salamanca, Salamanca, Spain, Paseo de San Vicente, 139, Salamanca, 37007 Spain; 6 Anesthesiology and Reanimation Service, Hospital Universitario de Salamanca, Salamanca, España; Paseo de San Vicente, 139, Salamanca, 37007 Spain; 7 General Surgery Service, Hospital Clínico Universitario de Valladolid, Avda Ramón y Cajal 3, 47005 Valladolid, Spain

## Abstract

Early recognition of sepsis is a key factor to improve survival to this disease in surgical patients, since it allows prompt control of the infectious source. Combining pro-inflammatory and immunosupression biomarkers could represent a good strategy to improve sepsis detection. Here we evaluated the combination of procalcitonin (PCT) with gene expression levels of HLA-DRA to detect sepsis in a cohort of 154 surgical patients (101 with sepsis and 53 with no infection). HLA-DRA expression was quantified using droplet digital PCR, a next-generation PCR technology. Area under the receiver operating curve analysis (AUROC) showed that the PCT/HLA-DRA ratio outperformed PCT to detect sepsis (AUROC [CI95%], *p*): PCT: 0.80 [0.73–0.88], <0.001; PCT/HLA-DRA: 0.85 [0.78–0.91], <0.001. In the multivariate analysis, the ratio showed a superior ability to predict sepsis compared to that of PCT (OR [CI 95%], *p*): PCT/HLA-DRA: 7.66 [1.82–32.29], 0.006; PCT: 4.21 [1.15–15.43] 0.030. Multivariate analysis was confirmed using a new surgical cohort with 74 sepsis patients and 21 controls: PCT/HLA-DRA: 34.86 [1.22–995.08], 0.038; PCT: 5.52 [0.40–75.78], 0.201. In conclusion, the combination of PCT with HLA-DRA is a promising strategy for improving sepsis detection in surgical patients.

## Introduction

Sepsis is a leading cause of morbidity and mortality in surgical patients, who account for nearly one third of all sepsis cases^1^. Sepsis is ten times more common than myocardial infarction and pulmonary embolism in these patients, and its associated mortality rate raises up to 39% if septic shock is present^2^. Success of treatment in sepsis is time-dependant^3^. Prompt administration of antibiotics decreases morbidity and mortality in a significant manner^4^. In surgical sepsis, to early find and control the source of infection is critical^5^. Nonetheless, identification of sepsis in surgical patients remains challenging^5,6^. In these patients, some of the early signs of sepsis pass unnoticed or are confused with signs of alarm of other conditions: inflammation due to infection overlaps with that secondary to surgery; altered mental status is often attributed to the administration of narcotic pain medication, oliguria is often attributed to under-resuscitation, hyperthermia frequently is not recognized as an early sign of sepsis, hypoxia in many occasions leads to think in pulmonary embolism but not in sepsis^5^.

Using biomarkers which reflect the patient’s response to the infection could help to improve sepsis diagnosis in surgical patients^7^. Sepsis is characterized by the presence of a dysregulated host response to infection. Between the features of this dysregulated response, sepsis is characterized by the simultaneous presence of exacerbated inflammation and depressed expression of molecules participating in the immunological synapse between antigen presentation cells and T lymphocytes, a key step for the development of a homeostatic adaptive response to infection^8^. In consequence, combining pro-inflammatory biomarkers with those reflecting immunosupression could represent a good strategy to increase the chances of detecting sepsis patients^9–11^.

Procalcitonin (PCT) is a precursor of the hormone calcitonin and is synthesized physiologically by thyroid C cells. In bacterial infections, systemic PCT secretion is a component of the inflammatory response, being in this context synthesized in various extrathyroidal neuroendocrine tissues^7^. Interpreted in combination with medical history, physical examination, and microbiological assessment, PCT could be a helpful biomarker for early diagnosis of sepsis^12^. Although many biomarkers aimed to the diagnosis of sepsis have been evaluated in the literature, PCT is still one of the most employed ones to rule in the presence of infection in the clinical practice. In turn, measuring gene expression levels in blood of HLA-DRA (encoding the non-polymorphic region of the alpha-chain of the HLA-DR molecule) has been proposed as a promising tool for evaluating the degree of immunosupression associated to sepsis^13,14^, and significantly predicts mortality in this disease^15^. In the present work, we employed droplet digital PCR (ddPCR)^16^, a next-generation polymerase chain reaction, to quantify gene expression of HLA-DRA in blood.

The objective of this study was to evaluate the accuracy of the combination between PCT levels in plasma and expression levels in blood of HLA-DRA to differentiate surgical patients with sepsis from those with no sepsis.

## Results

### Clinical characteristics of patients in the derivation cohort

patients with sepsis were mostly elderly male with co-morbidities, with high blood pressure, chronic cardiovascular disease, diabetes mellitus and antecedent of cancer being the most common. Abdominal surgery was the most frequent kind of surgery in this cohort. Of the 62 patients suffering from abdominal surgery, 46 patients showed an abdominal source of infection, 3 patients showed an infection coming from the surgical site, 2 patients had an urological infection as source of sepsis, 2 patients had a respiratory source, 1 had a Fournier gangrene, and the remaining 8 had an unidentified focus of infection. Necessity of urgent surgery was more frequent in the sepsis patients. Sepsis was of nosocomial origin in 60 cases. In these cases, sepsis followed surgery. In 41 cases, sepsis was originated in the community, representing the cause of surgery. Sepsis patients were more severe, as evidenced by the SOFA score at presentation to the hospital. Sepsis patients stayed longer at the ICU and also at the hospital. Hospital mortality was 24.7% in the sepsis group being absent in the group of patients with no sepsis (Table 1).

**Table 1:** Clinical characteristics of the patients in the derivation cohort: continuous variables are represented as median, (interquartile range, IQR); categorical variables were represented as (%, n).

		Sepsis patients (n = 101)	Surg. controls (n = 53)	*p*
Characteristics	Age [years, median (IQR)]	72.00 (15)	64.00 (18)	0.006
	Male	65.35 (66)	71.70 (38)	n.s.
Comorbidities, % (n)	High Blood Pressure	55.45 (56)	60 (30)	n.s.
	Chronic cardiovascular disease	47 (47)	32.65 (16)	n.s.
	Chronic respiratory disease	14.85 (15)	21.15 (11)	n.s.
	Chronic renal failure	14.85 (15)	2 (1)	n.s.
	Chronic hepatic failure	2.97 (3)	5.88 (3)	n.s.
	Neurologic disease	4.95 (5)	3.77 (2)	n.s.
	Cerebrovascular disease	3.96 (4)	5.66 (3)	n.s.
	Diabetes mellitus	33.66 (34)	18.87 (10)	0.005
	Cancer	29.70 (30)	45.3 (24)	0.050
	Immunosuppression	17.84 (17)	13.89 (5)	n.s.
Surgery type, % (n)	Urgent surgery	63.36 (64)	4.76 (2)	<0.001
	Cardio-thoracic	30 (30)	39.62 (21)	n.s.
	Abdominal	62 (62)	43.40 (23)	n.s.
	Neurosurgery	1 (1)	1.89 (1)	n.s.
	Vascular	3 (3)	3.77 (2)	n.s.
	Urological/Renal	1 (1)	9.43 (5)	0.011
	Other	3 (3)	1.89 (1)	n.s.
Time course and outcome	Length of hospital stay [days, median (IQR)]	26.5 (23.5)	10.0 (7.0)	<0.001
	Length of ICU stay [days,median (IQR)]	14.0 (17.0)	4.0 (3.0)	<0.001
	SIRS, % (n)	100 (101)	56.60 (30)	<0.001
	Septic Shock, % (n)	61.39 (62)	n.a	n.a.
	Non-survivors at day 28, % (n)	19.80 (20)	0	<0.001
	Hospital mortality, % (n)	24.75 (25)	0	<0.001
Source of infection, % (n)	Unknown	14.85 (15)	n.a.	n.a.
	Respiratory tract	18.81 (19)	n.a.	n.a.
	Abdomen	45.55 (46)	n.a.	n.a.
	Urinary tract	4.95 (5)	n.a.	n.a.
	Surgical site	5.94 (6)	n.a.	n.a.
	Bacteremia	4.95 (5)	n.a.	n.a.
	Other	4.95 (5)	n.a.	n.a.
Microbiology, % (n)	Gram+	46.5 (47)	n.a.	n.a.
	Gram−	58.4 (59)	n.a.	n.a.
	Fungi	6.9 (7)	n.a.	n.a.
	Virus	2 (2)	n.a.	n.a.
	Polymicrobial	40.6 (41)	n.a.	n.a.
Measurements at diagnosis, [median (IQR)]	SOFA score	8 (6)	1 (3)	<0.001
	Total bilirubin (mg/dl)	0.75 (1.30)	0.77 (0.53)	n.s.
	Glucose (mg/dl)	152.0 (78.0)	135.0 (55.0)	n.s.
	Platelet count (cell/mm^3^)	180000 (194.75)	155000 (740000)	n.s.
	INR	1.30 (0.42)	1.23 (0.41)	n.s.
	ScvO2 (%)	73.10 (19.00)	68.80 (80.00)	n.s.
	CRP (mg/L)	232.30 (169.25)	57.70 (77.70)	<0.001
	Procalcitonin (ng/mL)	5.57 (17.51)	0.50 (0.97)	<0.001
	White Blood cells (cells/mm^3^)	14465 (10257.50)	12290 (4875.0)	0.023
	Neutrophils (cells/mm^3^)	12337 (9220.50)	10016 (4704.5)	0.017

ICU: intensive care unit; SIRS: systemic inflammatory response syndrome, SOFA: Sequential Organ Failure Score; CRP, C reactive protein; INR, international normalized ratio; ScvO2: Central venous oxygen saturation; n.s: not significant; n.a: not applicable.

### Clinical characteristics of patients in the validation cohort

Sepsis patients had a similar profile in this cohort, being mostly elderly male with high blood pressure, diabetes mellitus and cancer as the most frequent antecedents. As occurred in the derivation cohort, abdominal surgery was the most frequent kind of surgery. Urgent surgery was also more frequent in the group of sepsis patients. Sepsis was of nosocomial origin in 33 cases, with the remainder cases being originated in the community, representing the cause of surgery. SOFA score was higher in the group of sepsis patients, although the difference with controls was not significant (0.063). Sepsis patients stayed longer at the ICU and also at the hospital. 12.2% of the patients with sepsis died during hospitalization, for none in the group of patients with no sepsis **(**Table 2**)**.

**Table 2:** Clinical characteristics of the patients in the validation cohort: continuous variables are represented as median, (interquartile range, IQR); categorical variables were represented as (%, n).

		Sepsis patients (n = 74)	Surg controls (n = 21)	p value
Characteristics	Age [years, median (IQR)]	70.50 (20)	70.00 (12)	n.s.
	Male [% (n)]	58.10 (43)	61.90 (13)	n.s.
Comorbidities, % (n)	High Blood Pressure	45.94 (34)	61.90 (13)	n.s.
	Chronic cardiovascular disease	9.46 (7)	9.52 (2)	n.s.
	Chronic respiratory disease	5.41 (4)	4.76 (1)	n.s.
	Chronic renal failure	8.11 (6)	9.52 (2)	n.s.
	Chronic hepatic failure	2.70 (2)	0 (0)	n.s.
	Diabetes mellitus	14.86 (11)	19.04 (4)	n.s.
	Cancer	18.91 (14)	42.86 (9)	0.024
	Immunosuppression	9.46 (7)	4.76 (1)	n.s.
Surgery type, % (n)	Urgent surgery	79.73 (59)	9.52 (2)	<0.001
	Abdominal	47.30 (35)	23.81 (5)	n.s.
	Vascular	2.70 (2)	9.52 (2)	n.s.
	Urological/Renal	1.35 (1)	14.29 (3)	0.009
	Other	4.05 (3)	0 (0)	n.s.
Time course and outcome	Length of hospital stay [days, median (IQR)]	16 (17)	10 (10)	0.048
	Length of ICU stay [days,median (IQR)]	3 (5)	2 (2)	0.050
	SIRS, % (n)	100 (74)	23.81 (5)	0.003
	Septic Shock, % (n)	48.64 (36)	0 (0)	<0.001
	Hospital mortality, % (n)	12.16 (9)	0 (0)	n.s.
	Respiratory tract, % (n)	14.86 (11)	n.a.	n.a.
	Abdomen, % (n)	40.54 (30)	n.a.	n.a.
	Urinary tract, % (n)	5.41 (4)	n.a.	n.a.
	Surgical site, % (n)	25.68 (19)	n.a.	n.a.
	Bacteremia, % (n)	14.86 (11)	n.a.	n.a.
	Other, % (n)	10.81 (8)	n.a.	n.a.
Microbiology, % (n)	Positive culture	45.94 (34)	n.a.	n.a.
	Gram+	18.92 (14)	n.a.	n.a.
	Gram−	32.43 (24)	n.a.	n.a.
	Fungi	1.35 (1)	n.a.	n.a.
	Virus	0 (0)	n.a.	n.a.
	Polymicrobial	18.92 (14)	n.a.	n.a.
Measurements at diagnosis, [median (IQR)]	SOFA score	6 (7)	2.50 (5)	n.s.
	Total bilirubin (mg/dl)	0.82 (1.06)	0.83 (0.59)	n.s.
	Glucose (mg/dl)	160 (73)	162 (39)	n.s.
	Platelet count (cell/mm^3^)	135000 (191500)	209000 (146000)	n.s.
	INR	1.22 (0.33)	1.11 (1.20)	0.023
	CRP (mg/L)	216.84 (179.83)	103.34 (98.85)	<0.001
	Procalcitonin (ng/mL)	2.70 (9.35)	0.29 (0.71)	<0.001
	White Blood cells (cells/mm^3^)	14960 (10600)	11280 (8075)	n.s.
	Neutrophils (cells/mm^3^)	13358.50 (11962.50)	10341.00 (9362)	n.s.

ICU: intensive care unit; SIRS: systemic inflammatory response syndrome, SOFA: Sequential Organ Failure Score; CRP, C reactive protein; INR, international normalized ratio; n.s: not significant; n.a: not applicable.

### Concentration of PCT and HLA-DRA across groups in the derivation cohort

**Figure 1:**
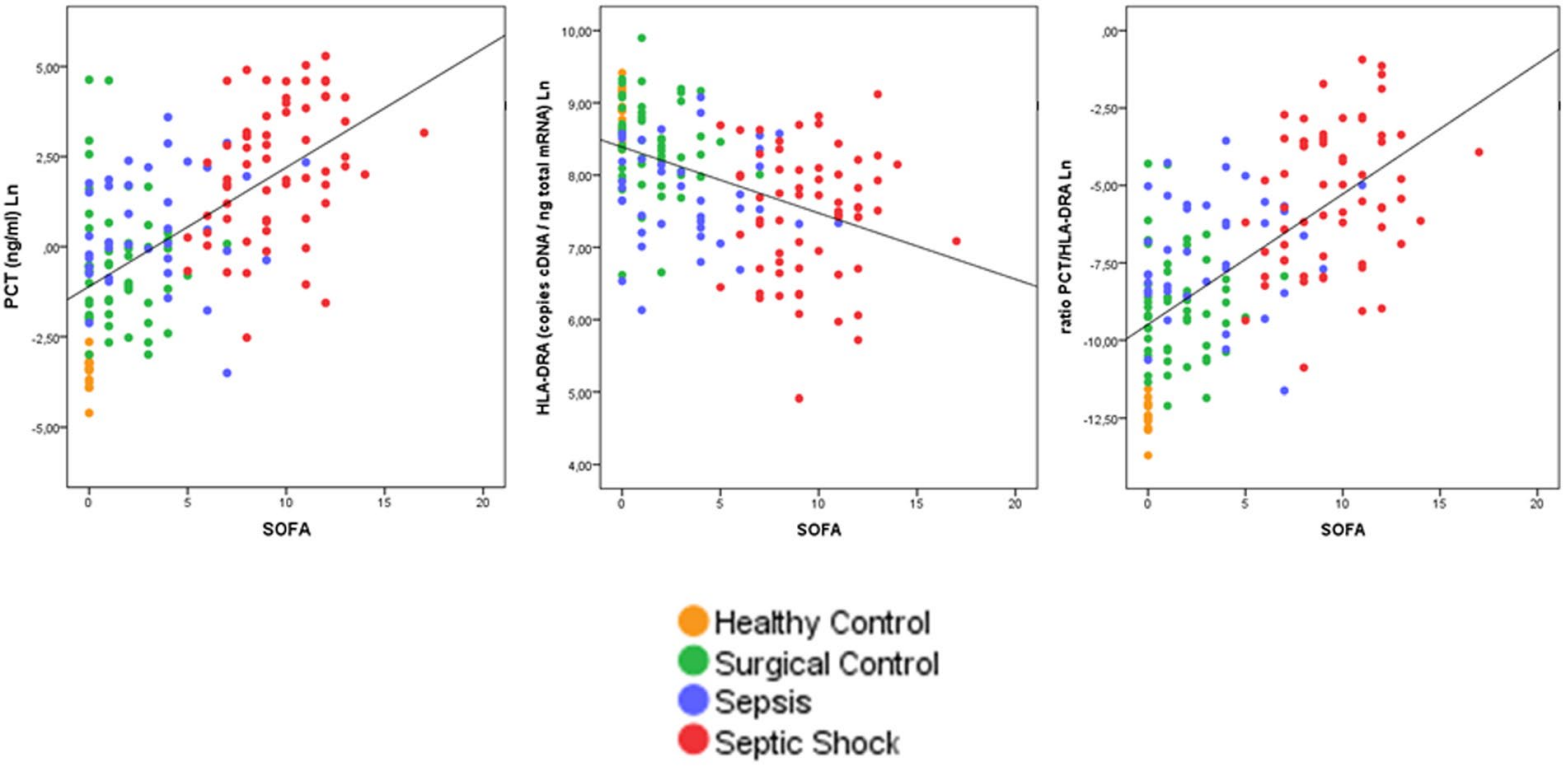
Dot plot showing patients‘ distribution depending on SOFA score and biomarkers‘ levels in the derivation cohort. Spearman correlation coefficients were 0.625, −0.488, 0.649 respectively (*p* < 0.001).

Median concentration of PCT (ng/mL) was as follows (median, interquartilic range in the group of healthy controls, surgical controls, sepsis patients with no septic shock and sepsis patients with septic shock respectively): 0.03 [0.02]; 0.50 [0.97]; 1.34 [5.74]; 9.55 [40.90]. In turn, HLA-DRA expression levels (cDNA copies/ng total mRNA) were as follows 7618 [2973]; 4468 [3884]; 2100 [2200]; 1858 [2415]. Finally, median values of the PCT/HLA-DRA ratio in the different groups were 0.0000 [0.000]; 0.0001 [0.000]; 0.0008 [0.000]; 0.0056 [0.030]. As showed in Fig. 1, PCT levels increased with organ failure degree and the presence of sepsis. In contrast, HLA-DRA gene expression levels decreased with organ failure degree and the presence of sepsis (Fig. 1). In consequence, the ratio between both biomarkers increased with disease severity and sepsis (Fig. 1). Levels of PCT and HLA-DRA showed an inverse and significant correlation (Supp File 1).

### Concentration of PCT and HLA-DRA across groups in the validation cohort

Median concentration of PCT (ng/mL) was as follows (median, interquartilic range in the group of surgical controls, sepsis patients with no septic shock and sepsis patients with septic shock respectively): 0.29 [0.71]; 1.03 [3.05]; 6.6 [25.7]. In turn, HLA-DRA expression levels (cDNA copies/ng total mRNA) were as follows 4928 [3456]; 2372 [4033]; 1866 [1560]. Finally, median values of the PCT/HLA-DRA ratio in the different groups were 0.0001 [0.000]; 0.0004 [0.000]; 0.0037 [0.020]. As occurred in the derivation cohort, the ratio correlated directly with the SOFA score (Spearman correlation coefficient (r) = 0.494, *p* < 0.001). Levels of PCT and HLA-DRA showed an inverse and significant correlation also (r = −0.490, *p* < 0.001).

### Accuracy for detecting sepsis

**Figure 2:**
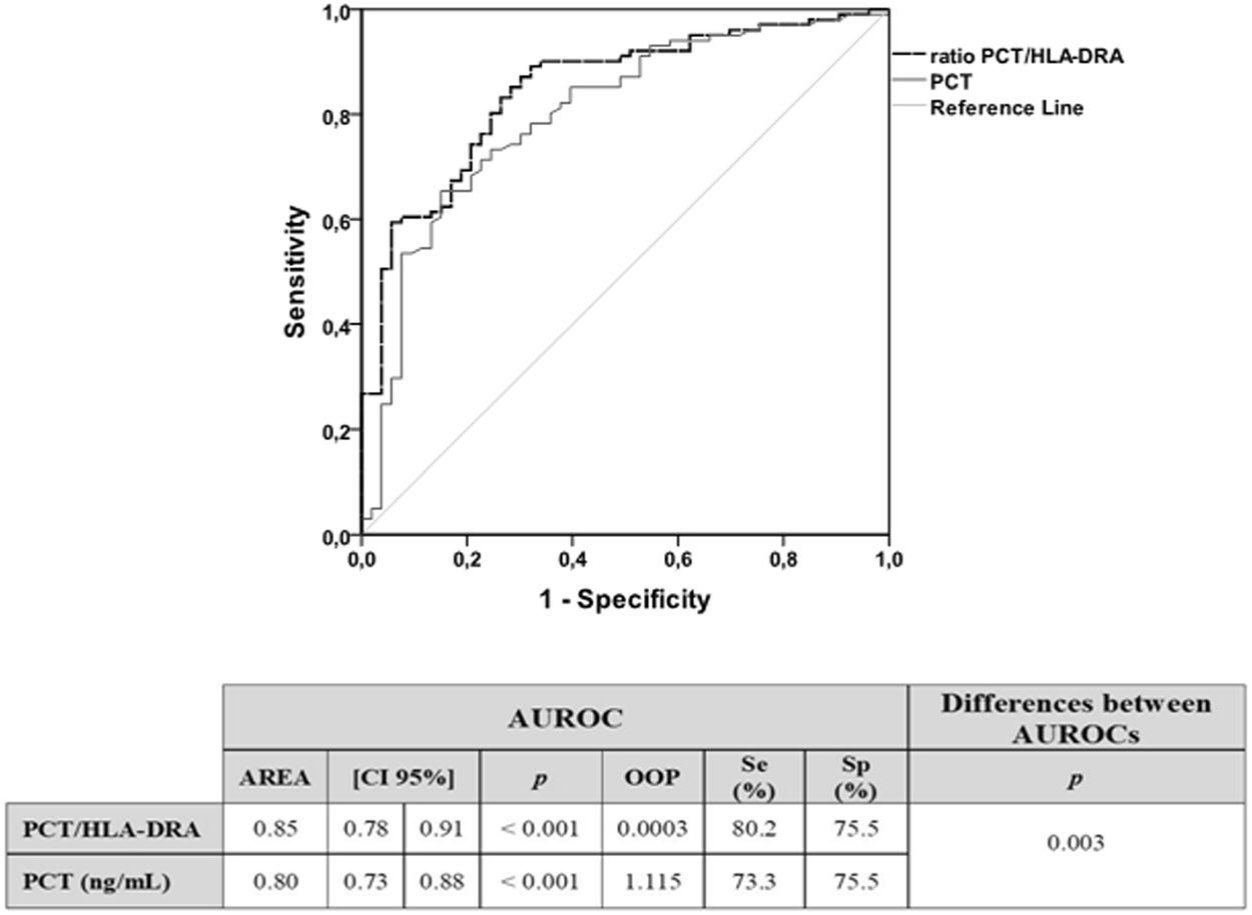
AUROC analysis for the differential diagnosis between sepsis patients and surgical controls with no infection in the derivation cohort. OOP: Optimal operating point; Se: sensitivity; Sp: Specificity.

The AUROC of PCT for differentiating between the group of sepsis patients and the group of surgical controls with no infection was compared against that obtained for the PCT/HLA-DRA ratio in the derivation cohort. As evidenced the DeLong test, the PCT/HLA-DRA ratio improved the diagnostic accuracy of PCT in a significant manner (Fig. 2). The superior diagnostic accuracy of the ratio compared to PCT was preserved when the analysis was repeated splitting sepsis patients in those with septic shock and those with no septic shock (Supp File 2). The optimal operating point (OOP) for PCT and the ratio was identified in each respective AUROC of Fig. 2. Sensitivity and specificity for each OOP are showed in this figure. Multivariate analysis demonstrated that the PCT/HLA-DRA ratio outperformed PCT in predicting sepsis (Table 3). A further multivariate analysis in the validation cohort confirmed the superiority of the PCT/HLA-DRA ratio over PCT (Table 4).

**Table 3:** Multivariate analysis for evaluating the risk of sepsis in the derivation cohort based on the PCT/HLA-DRA ratio or Procalcitonin levels.

	Multivariate analysis for PCT/HLA-DRA ratio				Multivariate analysis for PCT			
	OR	[CI 95%]		*p*	OR	[CI 95%]		*p*
SOFA Score	1.47	1.16	1.87	0.002	1.49	1.17	1.90	0.001
Age	1.00	0.94	1.06	0.873	1.01	0.96	1.07	0.714
Urgent Surgery	45.70	7.02	297.33	<0.001	41.57	6.77	255.39	<0.001
Chronic Renal Failure	8.09	0.40	165.00	0.174	4.74	0.26	86.12	0.293
Cancer	1.08	0.29	4.00	0.908	1.24	0.33	4.60	0.748
Diabetes Mellitus	2.89	0.69	12.22	0.149	2.41	0.61	9.57	0.210
Neutrophil concentration in blood (cells/mm^3^)	1.00	1.00	1.00	0.467	1.00	1.00	1.00	0.436
C-Reactive Protein	1.00	1.00	1.01	0.026	1.00	1.00	1.01	0.021
PCT/HLA-DRA > OOP (0.0003)	7.66	1.82	32.29	0.006	—	—	—	—
PCT > OOP (1.115 ng/mL)	—	—	—	—	4.21	1.15	15.43	0.030

OOP: Optimal operating point.

**Table 4:** Multivariate analysis for evaluating the risk of sepsis in the validation cohort based on the PCT/HLA-DRA ratio or Procalcitonin levels.

	Multivariate analysis for PCT/HLA-DRA ratio				Multivariate analysis for PCT			
	OR	[CI 95%]		*p*	OR	[CI 95%]		*p*
SOFA score	0.94	0.71	1.25	0.690	1.00	0.77	1.30	0.992
Cancer	0.17	0.02	1.34	0.092	0.20	0.03	1.34	0.097
Urgent Surgery	45.68	2.80	744.81	0.007	31.87	2.95	344.67	0.004
Neutrophil concentration in blood (cells/mm^3^)	1.00	1.00	1.00	0.471	1.00	1.00	1.00	0.754
C Reactive Protein	1.00	0.98	1.02	0.941	1.01	0.99	1.02	0.503
PCT/HLA-DRA > OOP (0.0003)	34.86	1.22	995.08	0.038	—	—	—	—
PCT > OOP (1.115 ng/mL)	—	—	—	—	5.52	0.40	75.78	0.201

Optimal operating points (OOP) obtained in the derivation cohort were tested in the validation cohort.

### SEPSIS-3 criteria

The AUROC analysis was repeated in the derivation cohort using the new diagnostic criteria proposed by the SEPSIS-3 consensus^17^. In consequence, 14 sepsis patients who did not score at least 2 points in SOFA were excluded from this new analysis. Using the SEPSIS-3 criteria, the PCT/HLA-DRA ratio also yielded better results than PCT to differentiate sepsis (n = 87 patients) from non sepsis patients (n = 53 patients): PCT [0.82 (0.75–0.90), 0.038]; PCT/HLA-DRA ratio [0.87 (0.81–0.93), 0.031] [AUROC (CI95%), *p*] (*p* = 0.004 in the Delong Test).

### Detecting mortality in sepsis

In the derivation cohort, AUROC analysis for differentiating survivors from non survivors in the group of patients with sepsis showed the following results: [AUROC, (confidence interval 95%), *p*]: PCT [0.78, (0.68–0.77) <0.001]; PCT/HLA-DRA [0.79, (0.70–0.89), <0.001]. Similarly occurred in the validation cohort: PCT [0.79, (0.65–0.93) 0.005]; PCT/HLA-DRA [0.76, (0.61–0.91), 0.012].

## Discussion

Early recognition and timely treatment largely determine outcome of sepsis^18^. This is particularly important in surgical sepsis, where prompt intervention to control the source of infection is a central element to increase survival^19^. Our study, using a derivation-validation approach, demonstrates for the first time that combining quantification of PCT, a peptide reflecting the host pro-inflammatory response to the infection, with that of HLA-DRA, a transcript reflecting the immunological suppression occurring in sepsis, is a good strategy to enhance chances of detecting this disease in surgical patients. Lethality of severe sepsis demands a screening mechanism exhibiting high sensitivity^20^. In this regard, the OOP identified for the PCT/HLA-DRA ratio improved the sensitivity of PCT to identify sepsis patients, preserving specificity (Fig. 2). This could help to improve performance of PCT as a screening biomarker for sepsis in this context. The logistic regression analysis performed in the two cohorts confirmed the superiority of the PCT/HLA-DRA ratio over PCT in predicting the presence of sepsis. The association between this ratio and sepsis was independent of disease severity, of potential confounding co-morbidities and of the basal inflammatory status of the patient as assessed by the CRP levels in serum and the neutrophil count in blood.

Our study demonstrates that the PCT/HLA-DRA ratio outperforms PCT independently of the presence or absence of septic shock in the sepsis patients, although it yielded the largest differences in distinguishing sepsis patients with no septic shock from the uninfected surgical controls (Supp File 2). This is certainly the most difficult scenario to identify sepsis in the clinical practice, and it is in consequence where the ratio could help the most.

PCT quantification is widely implemented in medium-large clinical settings as a complementary test to detect bacterial infection, which is the most frequent cause of sepsis. Gene expression profiling has demonstrated to be a valuable tool to identify the presence of sepsis^21,22^, but its translation to clinical practice has faced the absence of appropriate technology which could provide accurate and fast results. In this sense, ddPCR is a next generation PCR method that comes to solve these problems, since it allows absolute, reproducible quantification of gene expression in less than 3 hours, making this technology very attractive for clinical applications^23,24^.

A strength of our results is that they were confirmed when the analysis was repeated using the new criteria for sepsis diagnosis proposed by the SEPSIS-3 consensus^17^, criteria which have nonetheless suffered a number of major criticisms^20,25^ and which are yet pending to be endorsed by the International Statistical Classification of Diseases and Related Health Problems, ICD-11)^26^. In addition, our work is pioneer in proposing simultaneously profiling of protein and mRNA-based biomarkers as a new avenue for enhancing chances of sepsis detection. A limitation of our work is the absence of flow cytometry analysis of HLA-DRA expression on mononuclear cells. Future works should evaluate the same combination of PCT and HLA-DRA, profiling the later on monocyte and B lymphocyte surface by using flow cytometry, which is a widely available tool in most hospitals. Finally, although testing the ability to predict mortality was not the goal of our work, our results show that the PCT/HLA-DRA ratio did not improve the accuracy of PCT to differentiate survivors from non survivors.

In conclusion, our results support the combined quantification of PCT with that of HLA-DRA mRNA as a promising strategy for improving sepsis detection in surgical patients.

## Methods

### Ethics approval

The study was approved by the respective Committees for Ethics in Clinical Research of the participating hospitals (Hospital Clínico Universitario de Valladolid, CE-HCUV), (Hospital Universitario Río Hortega de Valladolid, CE-HURH), and (Hospital Clínico Universitario de Salamanca, CE-CAUSA). Methods were carried out in accordance with current Spanish law for Biomedical Research (2007). Written informed consent was obtained from the patients´ relatives or their legal representative before enrolment.

### Patients

#### Derivation cohort

A total of 101 adult patients (>18 years old) hospitalized at the Surgery Service or at the Surgical ICU of the participant hospitals with sepsis (according to the definition proposed by the American College of Chest Physicians/Society of Critical Care Medicine Consensus Conference)^27^ were prospectively included in the study by the participant physicians from April 2013 to January 2016. A control group consisting of 53 surgical patients with no signs of infection was also recruited. Finally, 16 blood donors of similar age of patients were included in the study. A specific standard survey was employed to collect the clinical data, including medical history, physical examination and hematological, biochemical, radiological and microbiological investigations. Treatment decisions were not standardized for all patients but were made by the treating physician.

#### Validation cohort

A total of 74 adult patients (>18 years old) hospitalized at the Surgery Service or at the Surgical ICU of the participant hospitals with sepsis (according to the definition proposed by the American College of Chest Physicians/Society of Critical Care Medicine Consensus Conference)^27^ were prospectively included in the study by the participant physicians from January 2017 to January 2018. A control group consisting of 21 surgical patients with no signs of infection was also recruited. A specific standard survey was employed to collect the clinical data, including medical history, physical examination and hematological, biochemical, radiological and microbiological investigations. Treatment decisions were not standardized for all patients but were made by the treating physician.

### Gene expression analysis

A sample of 2.5 mL of blood was collected by using PaxGene (BD) venous blood vacuum collection tubes in the first 12 hours following diagnosis of sepsis or in the first 12 hours following surgery in the case of the surgical controls. Blood from healthy individuals was collected at the moment of donation. Total RNA was extracted from blood samples using the PAXgene Blood RNA System (PreAnalytix, Hombrechtikon, Switzerland). The evaluation of concentration and quality was performed by spectrometry (Nano-Drop ND1000, NanoDrop Technologies,Wilmington, DE) and RNA Experion Bioanalyzer (BioRad, CA). Only samples with good quality and concentration were tested by ddPCR. Expression of HLA-DRA was quantified by ddPCR (BioRad) using predesigned TaqMan Assay Primer/Probe Sets, (FAM labeled MGB probes, Thermo Fisher/Scientific-Life Technologies, Waltham, MA): HLA-DRA (Hs00219575_m1). cDNA was generated from each sample on a Techne TC-512 thermal cycler (Bibby-Scientific, Staffordshire, OSA, UK) starting from 1000 ng of mRNA by using iScript Advanced cDNA Synthesis Kit (BioRad, cat:1725038). The obtained volume of cDNA (20 uL) was further diluted (1/25), and 2.5 uL (5 ng of total mRNA) were employed for quantification of target gene expression according to the manufacturer instruction’s. Briefly, ddPCR was performed using the BioRad QX200 ddPCR system, ddPCR Supermix for Probes (no dUTP), and BioRad standard reagents for droplet generation and reading. End-point PCR with 40 cycles was performed by using C1000Touch Thermal Cycler (BioRad) after splitting each sample into approximately 20,000 droplets. Next, the droplet reader used at least 10,000 droplets to determine the percentage of positive droplets and calculation of copy number of cDNA per nanogram of initial mRNA.

### Procalcitonin and C-reactive Protein Quantification

Procalcitonin (PCT) measurement in plasma was performed by electrochemiluminescence immunoassay on a chemistry analyzer (Cobas 6000, Roche Diagnostics, Meylan, France) in samples collected in the first 12 hours following diagnosis of sepsis or in the first 12 hours following surgery in the case of the surgical controls; limit of detection 0.02 ng/mL. Serum C-reactive protein (CRP) was measured by particle enhanced immunoturbidimetric assay (e501 Module Analyzer, Roche Diagnostics); limit of detection 0.15 mg/dL.

Both samples for HLA-DRA and PCT quantification were stored at −80 °C to be profiled in the same moment with the same platforms to avoid bias due to multiplatform testing.

### Statistical Analysis

Differences in demographic and clinical characteristics between patient groups were assessed using the χ^2^ test for categorical variables and the Mann Whitney U test for continuous variables. The accuracy of biomarkers for detecting sepsis was studied by calculating the area under the receiver operating characteristic curve (AUROC). The AUROC obtained for the PCT/HLA-DRA ratio was compared with that obtained for PCT alone by using the DeLong test^28^. The optimal operating point (OOP) was calculated, being the value for which the point on the curve had the minimum distance to the upper left corner (where sensitivity = 1 and specificity = 1). By Pithagoras’ theorem this distance is:

$$OOP=\sqrt{{(1-sensitivity)}^{2}+{(1-specificity)}^{2}}$$

Categorical variables were created, using each OOP identified as the cut-off. The association between these variables and the risk of presenting sepsis was further evaluated by logistic regression analysis. Potential confounding factors were identified from Table 1 by using an univariate analysis. Those variables yielding *p* values < 0.1 were further introduced as adjusting variables in the multivariate analysis. Multivariate analysis was repeated in the validation cohort using the same approach to validate the performance of the cut-offs identified in the derivation cohort. The statistical analysis was performed by using the IBM SPSS 22.0 software (SPSS, Chicago, IL, USA), except the DeLong test that was performed using the NCSS 12 software (Kaysville, Utah, USA). AUROC analysis was also employed to evaluate biomarkers‘ accuracy to predict mortality.

## Electronic supplementary material

Supplementary information

## Data Availability

Original data are available under reasonable request for scientist working in the field of sepsis.

## References

[1] Angus DC (2001). Epidemiology of severe sepsis in the United States: analysis of incidence, outcome, and associated costs of care. Crit. Care Med..

[2] Moore LJ (2010). Sepsis in general surgery: the 2005–2007 national surgical quality improvement program perspective. Arch. Surg. Chic. Ill 1960.

[3] Hotchkiss RS (2016). Sepsis and septic shock. Nat. Rev. Dis. Primer.

[4] Seymour CW (2017). Time to Treatment and Mortality during Mandated Emergency Care for Sepsis. N. Engl. J. Med..

[5] Moore LJ, Moore FA (2013). Early diagnosis and evidence-based care of surgical sepsis. J. Intensive Care Med..

[6] Moore, L. J. *et al*. Validation of a screening tool for the early identification of sepsis. *J. Trauma* **66**, 1539–1546, discussion 1546–1547 (2009). 10.1097/TA.0b013e3181a3ac4b 19509612

[7] Kibe S, Adams K, Barlow G (2011). Diagnostic and prognostic biomarkers of sepsis in critical care. J. Antimicrob. Chemother..

[8] Bermejo-Martin JF (2016). Defining immunological dysfunction in sepsis: A requisite tool for precision medicine. J. Infect..

[9] Hotchkiss RS, Monneret G, Payen D (2013). Immunosuppression in sepsis: a novel understanding of the disorder and a new therapeutic approach. Lancet Infect. Dis..

[10] Hotchkiss RS, Coopersmith CM, McDunn JE, Ferguson TA (2009). The sepsis seesaw: tilting toward immunosuppression. Nat. Med..

[11] Andaluz-Ojeda D (2012). A combined score of pro- and anti-inflammatory interleukins improves mortality prediction in severe sepsis. Cytokine.

[12] Wacker C, Prkno A, Brunkhorst FM, Schlattmann P (2013). Procalcitonin as a diagnostic marker for sepsis: a systematic review and meta-analysis. Lancet Infect. Dis..

[13] Cajander S (2013). Preliminary results in quantitation of HLA-DRA by real-time PCR: a promising approach to identify immunosuppression in sepsis. Crit. Care Lond. Engl..

[14] Winkler MS (2017). Human leucocyte antigen (HLA-DR) gene expression is reduced in sepsis and correlates with impaired TNFα response: A diagnostic tool for immunosuppression?. PloS One.

[15] Cazalis M-A (2013). Decreased HLA-DR antigen-associated invariant chain (CD74) mRNA expression predicts mortality after septic shock. Crit. Care Lond. Engl..

[16] Almansa, R. *et al*. Quantification of Immune Dysregulation by Next-generation Polymerase Chain Reaction to Improve Sepsis Diagnosis in Surgical Patients. *Ann. Surg*, 10.1097/SLA.0000000000002406 (2017). 10.1097/SLA.0000000000002406 28692472

[17] Singer M (2016). The Third International Consensus Definitions for Sepsis and Septic Shock (Sepsis-3). JAMA.

[18] Sartelli M (2018). Raising concerns about the Sepsis-3 definitions. World J. Emerg. Surg. WJES.

[19] Sotto A (2002). Evaluation of antimicrobial therapy management of 120 consecutive patients with secondary peritonitis. J. Antimicrob. Chemother..

[20] Simpson SQ (2016). New Sepsis Criteria: A Change We Should Not Make. Chest.

[21] Sweeney TE, Shidham A, Wong HR, Khatri P (2015). A comprehensive time-course-based multicohort analysis of sepsis and sterile inflammation reveals a robust diagnostic gene set. Sci. Transl. Med..

[22] Sweeney TE, Khatri P (2017). Benchmarking Sepsis Gene Expression Diagnostics Using Public Data. Crit. Care Med..

[23] Taylor SC, Laperriere G, Germain H (2017). Droplet Digital PCR versus qPCR for gene expression analysis with low abundant targets: from variable nonsense to publication quality data. Sci. Rep..

[24] Hindson CM (2013). Absolute quantification by droplet digital PCR versus analog real-time PCR. Nat. Methods.

[25] Bermejo-Martin JF, Martín-Fernandez M, Almansa R (2018). Pre-sepsis: A necessary concept to complete the SEPSIS-3 picture?. J. Crit. Care.

[26] WHO Joint Task force for ICD-11. Report of meeting of the WHO Joint Task force for ICD-11. (2017).

[27] Bone RC (1992). Definitions for sepsis and organ failure and guidelines for the use of innovative therapies in sepsis. The ACCP/SCCM Consensus Conference Committee. American College of Chest Physicians/Society of Critical Care Medicine. Chest.

[28] DeLong ER, DeLong DM, Clarke-Pearson DL (1988). Comparing the Areas under Two or More Correlated Receiver Operating Characteristic Curves: A Nonparametric Approach. Biometrics.

