# Author Correction: Combined quantification of procalcitonin and HLA-DR improves sepsis detection in surgical patients

**DOI:** 10.1038/s41598-019-39976-8

**Published:** 2019-02-27

**Authors:** Raquel Almansa, Silvia Martín, Marta Martin-Fernandez, María Heredia-Rodríguez, Esther Gómez-Sánchez, Marta Aragón, Cristina Andrés, Dolores Calvo, Jesus Rico-Feijoo, Maria Carmen Esteban-Velasco, Luis Mario Vaquero-Roncero, Alicia Ortega, Estefania Gómez-Pesquera, Mario Lorenzo-López, Iñigo López de Cenarruzabeitia, Diana Benavides, Jaime López-Sanchez, Cristina Doncel, Carmen González-Sanchez, Esther Zarca, Alberto Ríos-Llorente, Agustín Diaz, Elisa Sanchez-Barrado, Juan Beltran de Heredia, Jose Maria Calvo-Vecino, Luis Muñoz-Bellvís, Jose Ignacio Gomez-Herreras, César Aldecoa, Eduardo Tamayo, Jesus F. Bermejo-Martin

**Affiliations:** 1 Group for Biomedical Research in Sepsis (Bio∙Sepsis), Hospital Clínico Universitario de Valladolid/IECSCYL, Avda Ramón y Cajal 3, 47005 Valladolid, Spain; 2 Anesthesiology and Reanimation Service, Hospital Universitario Río Hortega, Calle Dulzaina, 2, 47012 Valladolid, Spain; 3 Anesthesiology and Reanimation Service, Hospital Clínico Universitario de Valladolid, Avda Ramón y Cajal 3, 47005 Valladolid, Spain; 4 Clinical Analysis Service, Hospital Clínico Universitario de Valladolid, Avda Ramón y Cajal 3, 47005 Valladolid, Spain; 5 Department of General and Gastrointestinal Surgery, Hospital Universitario de Salamanca, Salamanca, Spain; 6 Instituto de Investigación Biomédica de Salamanca (IBSAL), Universidad de Salamanca, Paseo de San Vicente, 139, Salamanca, 37007 Spain; 7 Anesthesiology and Reanimation Service, Hospital Universitario de Salamanca, Salamanca, España, Paseo de San Vicente, 139, Salamanca, 37007 Spain; 8 General Surgery Service, Hospital Clínico Universitario de Valladolid, Avda Ramón y Cajal 3, 47005 Valladolid, Spain

Correction to: *Scientific Reports* 10.1038/s41598-018-30505-7, published online 10 August 2018

The Acknowledgements section in this Article is incomplete.

“We thank the Nursery team of the participant clinical services for their continuous support to our research program. We appreciate also the assistance of Maria Jesús Garcia Salgado, from the Biobanco Hospital Clínico Universitario de Salamanca with sample storing. We thank the “Instituto de Salud Carlos III” and “Consejeria de Sanidad de Castilla y Leon” for their financial support, grant numbers [EMER 07/050], [PI15/01451], [PI16/01156] and “Consejería de Educación de Castilla y Leon/Fondo social Europeo” for supporting the contract of Marta Martin-Fernandez”.

should read:

“We thank the Nursery team of the participant clinical services for their continuous support to our research program. We appreciate also the assistance of Maria Jesús Garcia Salgado, from the Biobanco Hospital Clínico Universitario de Salamanca with sample storing. We thank the “Instituto de Salud Carlos III” and “Consejeria de Sanidad de Castilla y Leon” for their financial support, grant numbers [EMER 07/050], [PI15/01451], [PI16/01156] and “Consejería de Educación de Castilla y Leon/Fondo social Europeo” for supporting the contract of Marta Martin-Fernandez. This research has been supported with funds from “Fondo Europeo de Desarrollo Regional, Una manera de hacer Europa”.

