# Supplementary material for: Combined quantification of procalcitonin and HLA-DR improves sepsis detection in surgical patients
Source: Sci Rep. 2018 Aug 10;8:11999. doi: 10.1038/s41598-018-30505-7 (PMC6086887; doi:10.1038/s41598-018-30505-7)
Supplement: Supplementary file 1 — Supplementary information [file 41598_2018_30505_MOESM1_ESM.pdf]

Combined quantification of procalcitonin and HLA-DR improves sepsis detection in surgical patients.

Raquel Almansa , Silvia Martín , Marta Martin-Fernandez, María Heredia-Rodríguez , Esther Gómez-Sánchez, Marta Aragón, Cristina Andrés, Dolores Calvo, Jesus Rico-Feijoo, Maria Carmen Esteban-Velasco,Luis Mario Vaquero-Roncero , Alicia Ortega , Estefania Gómez-Pesquera , Mario Lorenzo-López ,Iñigo López de Cenarruzabeitia , Diana Benavides, Jaime López-Sanchez, Cristina Doncel , Carmen González-Sanchez , Esther Zarca, Alberto Ríos-Llorente, Agustín Diaz, Elisa Sanchez-Barrado, Juan Beltran de Heredia, Jose Maria Calvo-Vecino , Luis Muñoz Bellvís, Jose Ignacio Gomez-Herreras, César Aldecoa, Eduardo Tamayo,and Jesus F. Bermejo-Martin

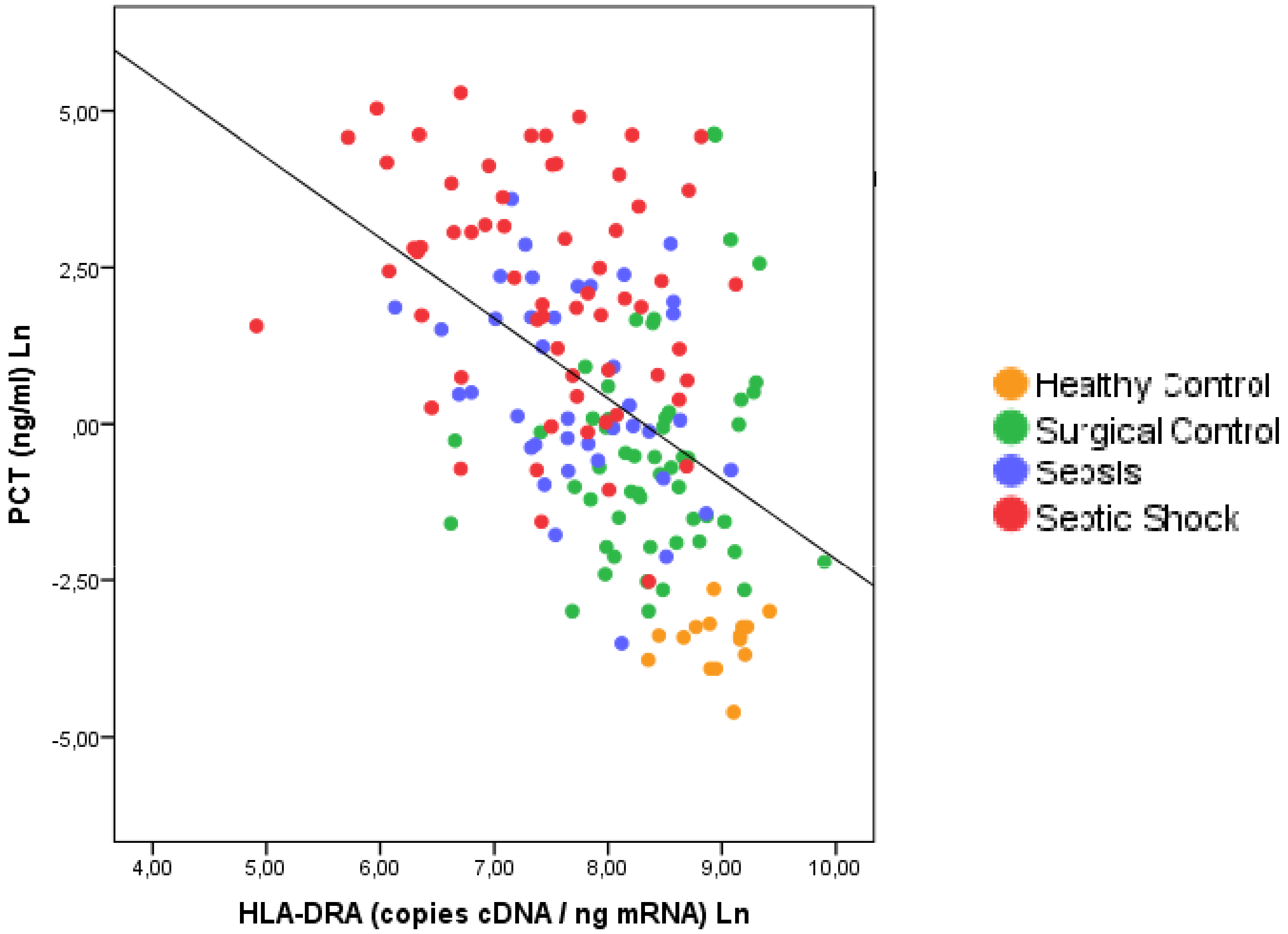

Supp file 1: Correlation between PCT and HLA-DRA gene expression levels in blood in the derivation cohort. Correlation Spearman coefficient = 0.466, $p < 0.001$.

A

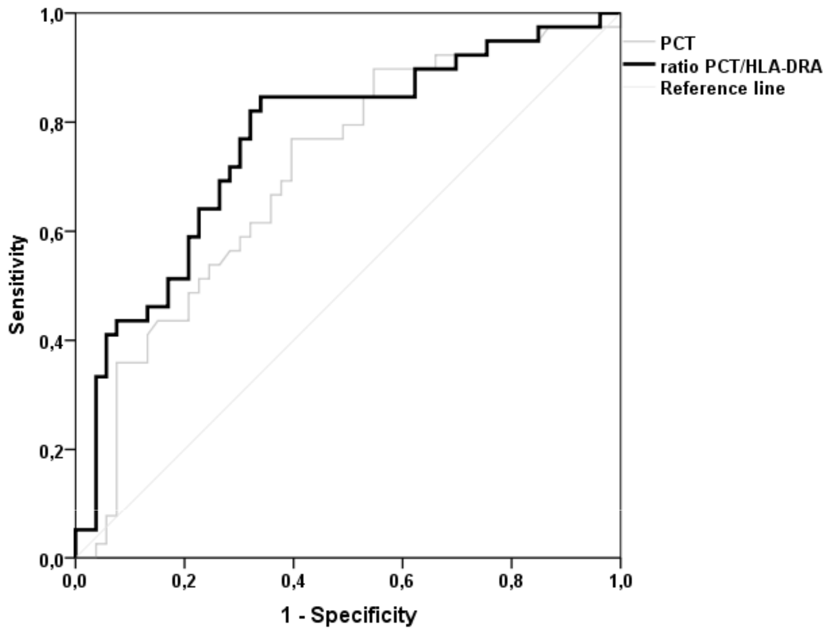

AUROC (CI-95%), *p*:
PCT: 0.71 (0.60 – 0.81), 0.001
PCT/HLA-DRA: 0.77 (0.66 – 0.86), < 0.001

B

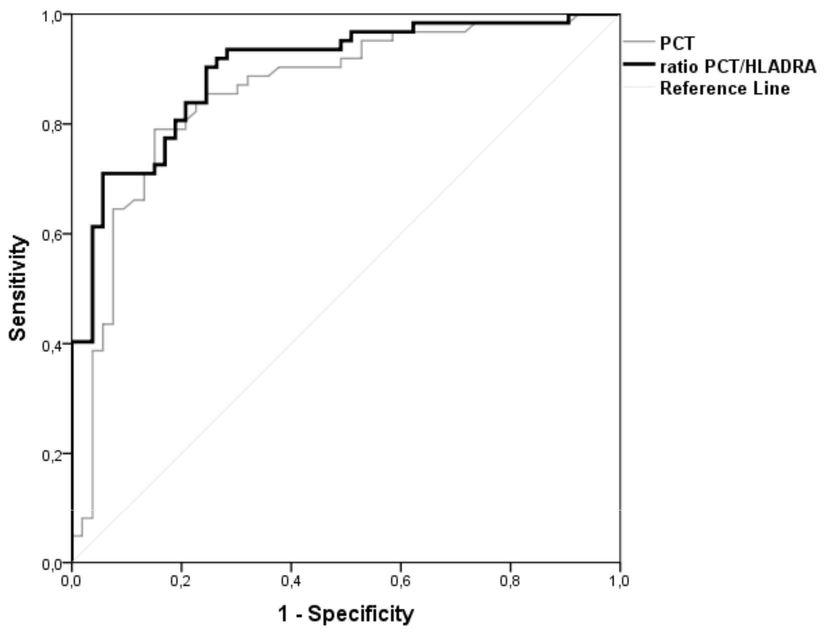

AUROC (CI-95%), *p*:
PCT: 0.86 (0.78 – 0.93), < 0.001
PCT/HLA-DRA: 0.90 (0.84 – 0.95), < 0.001

Supp file 2: A) AUROC analysis for the differential diagnosis between sepsis patients with no Septic Shock (n = 39) and surgical controls with no infection in the derivation cohort (n = 53). B) AUROC analysis for the differential diagnosis between sepsis patients with Septic Shock (n = 62) and surgical controls with no infection in the derivation cohort (n = 53).
